# Linc-ROR promotes esophageal squamous cell carcinoma progression through the derepression of SOX9

**DOI:** 10.1186/s13046-017-0658-2

**Published:** 2017-12-13

**Authors:** Lianghai Wang, Xiaodan Yu, Zhiyu Zhang, Lijuan Pang, Jiang Xu, Jinfang Jiang, Weihua Liang, Yuhang Chai, Jun Hou, Feng Li

**Affiliations:** 1 Department of Pathology and Key Laboratories for Xinjiang Endemic and Ethnic Diseases, Shihezi University School of Medicine, Shihezi, Xinjiang China; 2 Department of Immunology, Shihezi University School of Medicine, Shihezi, Xinjiang China; 3 Department of Pathology, Beijing Chaoyang Hospital, Capital Medical University, Beijing, China; 4 Department of Biomedical Sciences and Cornell Stem Cell Program, Cornell University, Ithaca, NY USA; 5 Department of Stomatology, The First Affiliated Hospital of Shihezi University School of Medicine, Shihezi, Xinjiang China

**Keywords:** Linc-ROR, SOX9, Stemness, Therapy

## Abstract

**Background:**

Novel therapies tailored to the molecular composition of esophageal squamous cell carcinoma (ESCC) are needed to improve patient survival. We investigated the regulatory network of long intergenic non-protein coding RNA, regulator of reprogramming (linc-ROR) and sex-determining region Y-box 9 (SOX9), and their therapeutic relevance in ESCC.

**Methods:**

Linc-ROR and SOX9 expression were examined in ESCC specimens, cell lines, and cultured tumorspheres. We investigated the effects of linc-ROR on SOX9 expression and malignant phenotypes by CCK8, colony formation, Transwell, and sphere-forming assay. The linc-ROR/SOX9 interaction mediated by multiple microRNAs (miRNAs) was confirmed by bioinformatic analysis, luciferase assay, and RNA-binding protein immunoprecipitation, transient overexpression or antagonizing endogenous candidate miRNAs. The effect of linc-ROR depletion on tumor growth was assessed by xenograft assay.

**Results:**

A positive correlation between linc-ROR and SOX9 expression was found in clinical ESCC specimens (*r* = 0.562, *P* = 0.036), cell lines, and tumorspheres. Silencing of linc-ROR significantly inhibited cell proliferation, motility, chemoresistance, and self-renewal capacity. Mechanistically, linc-ROR modulating the derepression of SOX9 by directly sponging multiple miRNAs including miR-15b, miR-33a, miR-129, miR-145, and miR-206. Antagonizing these miRNAs counteracted with linc-ROR silencing, whereas the repression of SOX9 abrogated malignant phenotypes induced by the cocktail of miRNA inhibitors. Moreover, linc-ROR disruption was sufficient to attenuate tumor growth and cancer stem cell marker expression in vivo.

**Conclusions:**

Our results demonstrate that the linc-ROR–miRNA–SOX9 regulatory network may represent a novel therapeutic target for ESCC.

**Electronic supplementary material:**

The online version of this article (10.1186/s13046-017-0658-2) contains supplementary material, which is available to authorized users.

## Background

Esophageal cancer is the sixth most common cause of cancer-associated mortality worldwide [1]. More than 480,000 patients are diagnosed with esophageal cancer, and 400,000 of these patients die from the disease annually [2]. Esophageal squamous cell carcinoma (ESCC) is one of the major histologic subtypes of esophageal cancer [3], particularly in high-incidence areas of East Asia and East Africa [4, 5]. Even with improved effectiveness of diagnosis and combination of surgical approach and chemoradiotherapy, the prognosis of esophageal cancer is still not ideal; population-based studies have shown the overall five-year survival rate is less than 20% in the past decade [6–8]. Thus, understanding the detailed molecular mechanisms in ESCC progression and developing novel therapeutic strategies are urgently needed to improve the survival rates of these patients.

Long intergenic non-protein coding RNA, regulator of reprogramming (linc-ROR) was first identified as a regulator for reprogramming of differentiated cells to induce pluripotent stem cells and maintenance of pluripotency in 2010 [9]. Increasing evidence indicate that linc-ROR also plays a role in tumorigenesis and tumor progression; in most cases, it acts as an oncogene in cancers. Marked upregulation of linc-ROR has been observed in various tumors, including cancers of the nasopharynx [10], breast [11], liver [12], gallbladder [13], pancreas [14], endometrium [15], and esophagus [16]. Recently, subsequent evidence indicate that linc-ROR may function as a competitive endogenous RNA (ceRNA), also known as “microRNA (miRNA) sponge”, to antagonize the regulatory function of miRNA [17–19]. However, the overall biological role and underlying mechanism of linc-ROR in ESCC remain largely unknown.

Sex-determining region Y-box 9 (SOX9) is a high-mobility group box-containing transcription factor that plays critical roles in embryogenesis, organ development, and maintenance of stem/progenitor cells [20–22]. Dysregulation of SOX9 has been further implicated in cancer progression as an oncogene, which promotes cell proliferation, inhibits senescence, and facilitates transformation [23]. In addition, high level of SOX9 expression was reported to confer the properties associated with cancer stem cell (CSC) and correlate with epithelial–mesenchymal transition (EMT) through triggering signaling cascades including the WNT/β-catenin pathway [24–29]. Elevated SOX9 expression has also been observed in esophageal cancer cell lines and clinical specimens, correlating with poor prognosis in patients with ESCC and conferring CSC properties including tumorsphere formation and tumorigenicity in esophageal adenocarcinoma [25, 30]. Taken together, these studies imply that linc-ROR and SOX9 are involved in stem/progenitor cell maintenance, but their roles in regulating cancer development remains to be explained.

In this study, we investigated the biological roles of linc-ROR on the malignant phenotypes of ESCC cells in vitro and in vivo. Mechanistic analysis revealed that linc-ROR deregulate the expression of pluripotency transcription factor SOX9 through competition with multiple miRNAs, including miR-15b, miR-33a, miR-129, miR-145 and miR-206, thus playing an oncogenic role in ESCC. The present work provides the first evidence for a positive linc-ROR/SOX9 correlation mediated by multiple miRNAs, which may shed a new light on the treatment of ESCC.

## Methods

### Cell lines and human tissue samples

ESCC cell lines Eca109, EC9706, KYSE150, and TE-1 and embryonic kidney cell line 293 T were purchased from the Cell Bank of Type Culture Collection of Chinese Academy of Sciences (Shanghai Institute of Biochemistry and Cell Biology). All cells were maintained at 37 °C in a humidified incubator under 5% CO_2_ atmosphere.

Fourteen pairs of primary ESCC and adjacent non-tumor esophageal tissues were obtained from patients undergoing esophagectomy without prior radiotherapy or chemotherapy at Kashgar Hospital from 2013 to 2015. This study was approved by the Ethics Committee of Shihezi University School of Medicine, and all participants were enrolled with written informed consent. Clinical characteristics of all patients are summarized in Additional file 1 Table S1.

### Sphere-forming assay

Eca109 cells were seeded at 1.5 × 10^3^ cells/well in six-well ultra-low cluster plates (Corning) and cultured in DMEM/F12 serum-free medium (Gibco) supplemented with 2% B27 (Invitrogen), 20 ng/mL EGF (PeproTech), and 20 ng/mL bFGF (PeproTech) for 10 days. Subsequently, tumorspheres with diameter larger than 75 μm were counted.

### siRNA and miRNA transfection

siRNA against human linc-ROR (coding region sense was 5′-GGAGAGGAAGCCUGAGAGUdTdT-3′, antisense was 5′-ACUCUCAGGCUUCCUCUCCdTdT-3′) and SOX9 coding region sense was 5′-CGCUCACAGUACGACUACAdTdT-3′, antisense was 5′-UGUAGUCGUACUGUGAGCGdTdT-3′), miRNA mimics, antisense miRNA inhibitors, and negative scramble control RNA oligos were synthesized by GenePharma. RNA transfection was performed at a final concentration of 50 nM, using Lipofectamine 2000 Transfection Reagent (Invitrogen) according to the manufacturer’s instructions.

### RNA isolation and quantitative real-time PCR

Total RNA was isolated from cultured cells or human samples using Total RNA Kit I (Omega Bio-tek) or miRNeasy FFPE Kit (Qiagen) according to the manufacturer’s protocols, respectively. cDNA was synthesized using reverse transcriptase, after which quantitative real-time PCR (qRT-PCR) was performed using Fast SYBR qPCR mixture (CWBIO) with specific primers on 7500 Fast Real-Time PCR System (Applied Biosystems). GAPDH or U6 was used as an internal control. Primer sequences are listed in Additional file 1 Table S2.

### Cell proliferation assay

Cell proliferation was measured with Cell Counting Kit-8 (CCK-8) (Dojindo). Different groups of cells were plated at 3 × 10^3^ cells/well in 96-well plates. At 0, 24, 48, and 72 h post-plating, CCK-8 solution was added to each well, and the absorbance at 450 nm (OD_450_) was measured after incubation for 40 min at 37 °C.

### Colony formation assay

After 24 h transfection with various RNA oligos, cells were seeded in six-well plates and cultured for 10 days. After fixation with methanol for 15 min, the colonies were stained with 0.1% crystal violet in 20% methanol and counted.

### Migration and invasion assays

Migration and invasion assays were carried out using a Transwell chamber (Corning). Cells with serum-free medium were seeded in the top chamber without or with an insert coated with Matrigel (BD Biosciences) for migration assay and invasion assay, respectively. The lower chamber was filled with medium with 20% FBS as chemoattractant. After incubation for 24 h, the cells that have migrated or invaded through the membrane and the cells on the lower surface of the member were fixed, stained, and counted.

### Dual luciferase reporter assay

293 T cells were seeded at 3 × 10^4^ cells/well in 24-well plates and allowed to settle for 24 h. Subsequently, cells were transfected with various RNA oligos together with pmiR-REPORT-SOX9 3′-untranslated region (3′-UTR) reporter plasmid and a Renilla luciferase vector. Forty-eight hours after transfection, relative luciferase activity was measured using the Dual-Luciferase Reporter Assay System (Promega) and normalized against Renilla luciferase activity.

### RNA-binding protein immunoprecipitation

RNA-binding protein immunoprecipitation (RIP) experiments were performed using 10 μg Ago2 antibody (ab5072; Abcam) and the Magna RIP Kit (Millipore) in accordance to the manufacturer’s instructions. Co-precipitated RNAs were then analyzed using qRT-PCR analysis.

### Western blot

Equal amounts of cell lysates were electrophoretically separated and transferred to a PVDF membrane. After blocking with 5% skimmed milk, the membrane was incubated with primary antibodies against SOX9 (AB5535, 1:2000; Millipore), E-cadherin (sc-8426, 1:100; Santa Cruz), vimentin (sc-6260, 1:100; Santa Cruz), β-actin (sc-7963, 1:100; Santa Cruz), and appropriate secondary antibodies. The signals were detected using enhanced chemiluminescence (GE Healthcare).

### ESCC xenografts and siRNA treatment

All animal experiments were approved by the Research Ethics Committee of the First Affiliated Hospital of Shihezi University School of Medicine. Five-week-old female athymic BALB/C nude mice were purchased from Beijing Vital River Laboratory Animal Technology. ESCC xenografts were established in the flanks by subcutaneously inoculating 1 × 10^6^ EC9706 cells. Mice bearing established xenografts were treated with 5 nM of cholesterol-conjugated linc-ROR siRNA (Chol-silinc-ROR) or negative control with 100 μL PBS by locally injecting into the tumor mass every 3 days for a total of six times. Three days after the last injection, the mice were sacrificed, and the tumors were excised and weighed. Tumor volumes were calculated according to the formula: length × width^2^/2. Subsequently, tumors were fixed and paraffin embedded. Sections of 4 μm were cut and subjected to hematoxylin–eosin or immunohistochemistry staining [31] using anti-SOX9 (AB5535, 1:600; Millipore), anti-CD44 (EP44, 1:50; ZSGB-BIO), and anti-vimentin (OTI5D7, 1:600; ZSGB-BIO) antibodies. Negative controls were prepared by replacing the primary antibodies with PBS.

### Statistical analysis

Statistical analyses were performed using SPSS 12.0 software and GraphPad Prism 5.0. Comparison between groups was conducted using two-tailed Student’s t-test or one-way ANOVA followed by post hoc tests. Pearson correlation was used to evaluate the significance of association between linc-ROR and SOX9 levels. Numerical data were presented as means ± SEM unless stressed. Statistical significance was considered at *P* < 0.05.

## Results

### Linc-ROR is positively correlated with SOX9 expression in ESCC

**Fig. 1:**
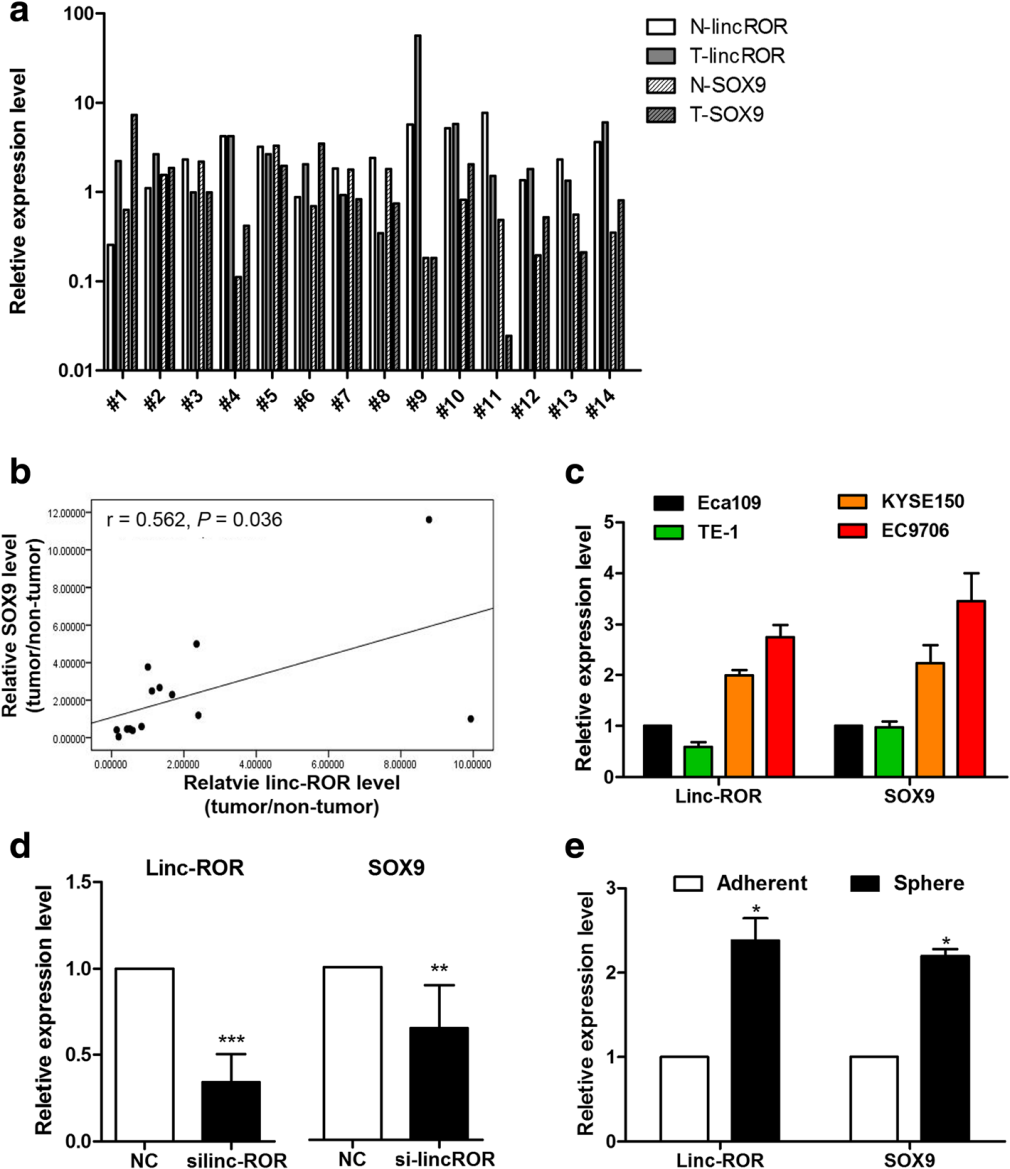
Linc-ROR is positively correlated with SOX9 expression in ESCC. **a** Expression of linc-ROR and SOX9 was analyzed in 14 paired ESCC (T) and corresponding non-tumor esophageal tissues (N) using qRT-PCR. Transcription levels were normalized to GAPDH expression. **b** Correlation between the relative expression levels of linc-ROR and SOX9 in paired ESCC specimens. **c** Expression levels of linc-ROR and SOX9 in ESCC cell lines were determined by qRT-PCR. **d** Assessment of the expression of linc-ROR and SOX9 after EC9706 cells were treated with non-specific siRNA control (NC) or siRNA against linc-ROR (silinc-ROR) by qRT-PCR. **e** Quantification of the expression of linc-ROR and SOX9 in Eca109 cells on monolayer or three-dimensional cultures

To determine whether linc-ROR expression correlates with SOX9 expression in ESCC, their gene expression levels were determined in 14 paired ESCC and adjacent esophageal tissue samples (Fig. 1a). Interestingly, a significant positive correlation was observed between the relative expression of linc-ROR and SOX9 in ESCC samples when compared to their matched non-tumor tissues (*r* = 0.562, *P* = 0.036; Fig. 1b). Subsequently, we examined the expression levels of linc-ROR and SOX9 in a panel of ECSS cell lines (Eca109, EC9706, KYSE150, and TE-1). Cell lines (EC9706 and KYSE150) with higher expression of linc-ROR also exhibited higher levels of SOX9 (Fig. 1c). Accordingly, we found that knockdown of endogenous linc-ROR by siRNA resulted in a significant reduction of SOX9 expression in EC9706 cells (Fig. 1d). Consistent with these observations, the expression of linc-ROR, as well as the known stem/progenitor cell marker SOX9 [32], were higher when Eca109 cells were grown as tumorspheres (stem like) in three-dimensional suspension cultures than when grown as two-dimensional adherent (differentiated) cultures (Fig. 1e). Collectively, these results indicated a positive correlation between linc-ROR and SOX9 expression in ESCC.

### Inhibition of linc-ROR decreases CSC-like properties

**Fig. 2:**
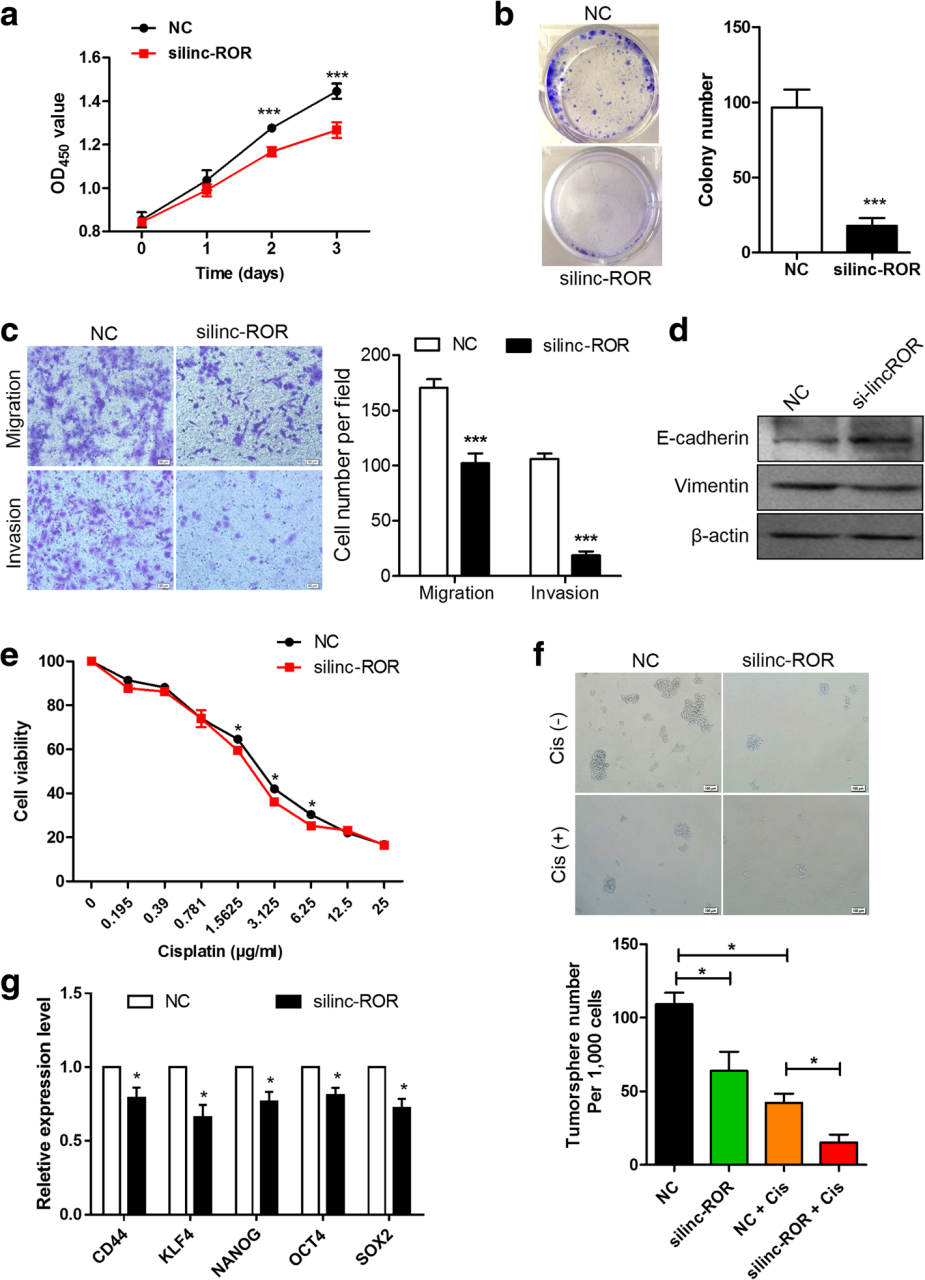
Inhibition of linc-ROR decreases CSC-like properties. **a** CCK8 assay revealed a time-dependent inhibition of cell proliferation after linc-ROR siRNA treatment compared with scramble control (NC) in EC9706 cells. **b** Reduced colony-forming capacity of EC9706 cells after silencing of linc-ROR. **c** Effect of linc-ROR knockdown on cell migration (top) and invasion (bottom) of EC9706 cells assessed using Transwell assay. **d** Immunoblot analysis of E-cadherin and vimentin expression in EC9706 cells after treatment with linc-ROR siRNA. β-actin was used as a loading control. **e** Cell viability of EC9706 after treatment with scramble or linc-ROR siRNA was determined by CCK8 assay in the presence of indicated doses of cisplatin. **f** Representative images of tumorspheres (top) and quantification of tumorsphere numbers (bottom) formed by Eca109 cells (per 1000 cells) after linc-ROR knockdown in the presence or absence of cisplatin (Cis; 3 μg/mL). **g** qRT-PCR analysis of stemness-associated genes CD44, KLF4, NANOG, OCT4, and SOX2 expression in EC9706 cells following linc-ROR knockdown. Transcription levels were normalized to GAPDH expression

The observation that linc-ROR and SOX9 are coordinately expressed in ESCC tissues led to the hypothesis that linc-ROR might promote stem cell-like properties and cancer progression through the regulation of pluripotency transcription factor SOX9. To determine this possibility, we first transfected EC9706 cells with siRNA to efficiently deplete the endogenously expressed linc-ROR. The growth curves detected by CCK8 showed that linc-ROR repression significantly decreased cell proliferation (Fig. 2a). Linc-ROR downregulation also substantially repressed colony-forming capacity as indicated by the formation of fewer and smaller colonies (Fig. 2b). As enhanced cancer cell motility and consequent invasion and metastasis have been associated with the gain of CSC properties and EMT [33], we investigated the effect of linc-ROR inhibition on these phenotypes. Knockdown of linc-ROR in EC9706 cells resulted in a significant attenuation of their migration ability and invasive potential compared to control groups using a Transwell system (Fig. 2c). Accordingly, the expression of epithelial cell marker E-cadherin was robustly increased in silinc-ROR-transfected cells, whereas the expression of mesenchymal marker vimentin was significantly reduced (Fig. 2d), indicating that linc-ROR can promote EMT in ESCC cells and hence facilitates their mobility. Chemoresistance is one of the major characteristics of CSC [34]. We found that linc-ROR repression resulted in the sensitization of EC9706 cells to cisplatin, which is one of the most frequently used chemotherapeutic drug for esophageal cancer [6] (Fig. 2e). With non-adherent sphere formation assay, the tumorsphere-forming capacity was significantly decreased following inhibition of linc-ROR in Eca109 cells compared to scramble control (Fig. 2f). Furthermore, the combination of linc-ROR knockdown and cisplatin treatment synergistically reduced the number and the size of tumorspheres compared to that of cisplatin treatment alone. The expression of stemness-associated genes, including CD44, KLF4, NANOG, OCT4, and SOX2 was also markedly decreased by linc-ROR knockdown in concert with the reduced sphere-forming ability (Fig. 2g). These results suggest that linc-ROR promotes the acquisition of CSC-like properties in ESCC.

### Linc-RoR shares miRNA binding sites with Sox9

**Fig. 3:**
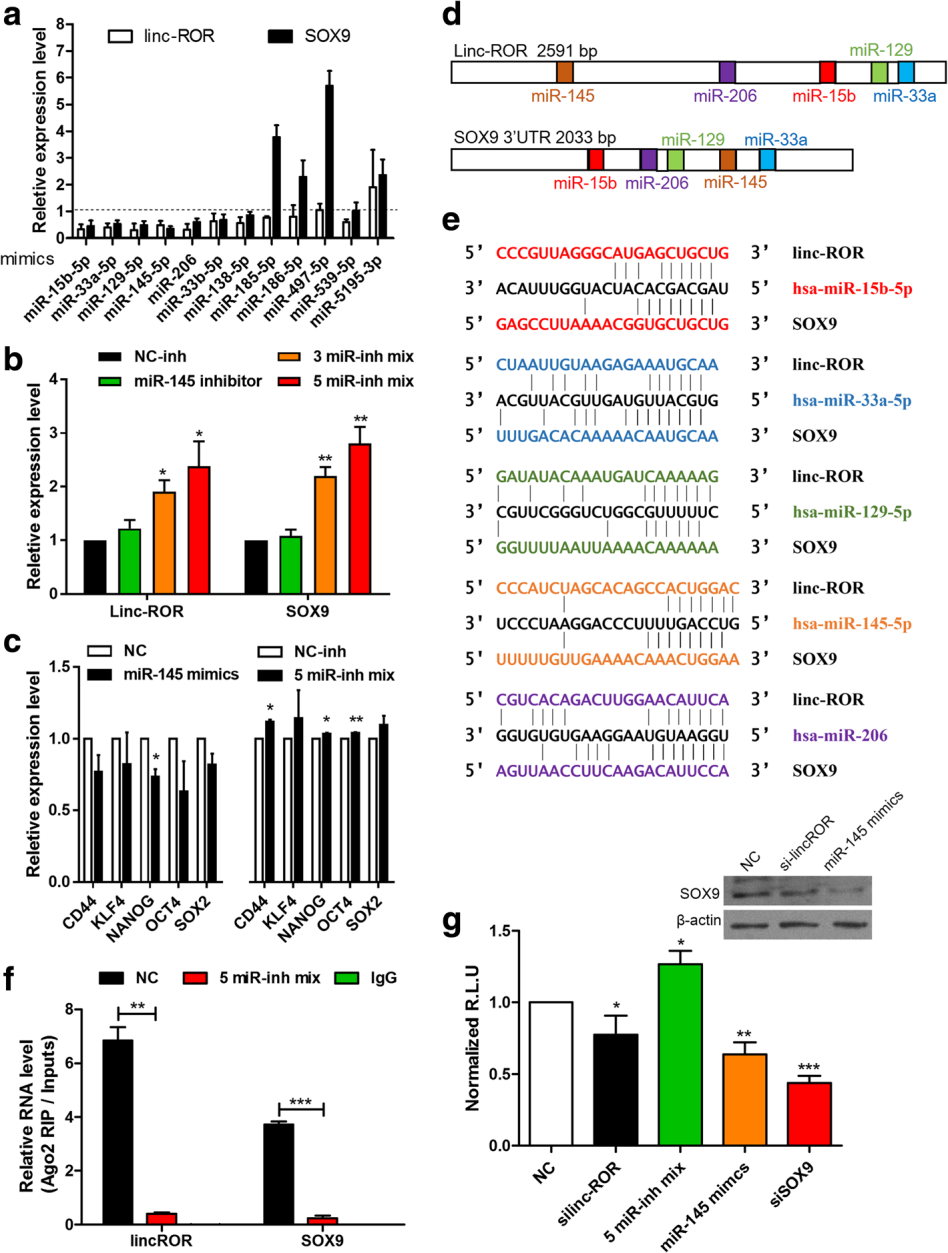
Linc-ROR shares miRNA binding sites with SOX9. **a** qRT-PCR analysis of the expression of linc-ROR and SOX9 in EC9706 cells transfected with 12 different miRNA mimics versus scramble control. **b** qRT-PCR analysis of linc-ROR and SOX9 expression after treatment with miR-145 inhibitor; a mixture of miR-15b, miR-33a, and miR-129 inhibitors (3 miR-inh mix); and inhibitor cocktail of miR-15b, miR-33a, miR-129, miR-145, and miR-206 (5 miR-inh mix). **c** qRT-PCR analysis of stemness-associated genes CD44, KLF4, NANOG, OCT4, and SOX2 expression following treatment with miR-145 mimics in EC9706 or 5 miR-inh mix in Eca109 cells. Transcription levels were normalized to GAPDH expression. **d** Prediction for five candidate miRNA-binding elements on linc-ROR transcript and SOX9 3′-UTR. **e** Sequence alignment of miR-15b, miR-33a, miR-129, miR-145, and miR-206 seed sequence in linc-ROR and SOX9 3′-UTR. **f** Amount of linc-ROR and SOX9 bound to Ago2 was determined by qRT-PCR in the presence of inhibitor cocktail of all candidate miRNAs or negative control. IgG was used as a negative control. **g** SOX9 protein level in EC9706 cells following treatment with linc-ROR siRNA or miR-145 mimics was measured by Western blot (top). Luciferase assay of 293 T cells co-transfected with pmiR-REPORT-SOX9 3′-UTR and indicated RNA oligos. siRNA targeting SOX9 3′-UTR was used as a positive control (bottom)

Increasing evidence suggests that linc-ROR participates in the regulatory network of ceRNA [19, 35]. We hypothesized that endogenous linc-RoR also function as a ceRNA of SOX9 in ESCC. We used a target prediction tool based on RNA22 [36] to search for miRNAs that target the full-length transcripts of linc-RoR and bioinformatics tools miRWalk 2.0 [37] and miRanda [38] to search for miRNAs that target the 3′-UTR of SOX9 mRNA. Twelve miRNAs whose predicted binding sites were shared by linc-RoR and SOX9 emerged (Additional file 1 Table S3). Mimics of these miRNAs were transfected into EC9706 cells; miR-15b, miR-33a, miR-129, miR-145, and miR-206 exhibited the most significant inhibitory effect on the expression of linc-ROR and SOX9 (Fig. 3a). To assess the potential relationship between linc-ROR, SOX9, and these miRNAs, we transfected Eca109 cells with the inhibitor of miR-145; the inhibitor mixture of miR-15b, miR-33a, and miR-129; and inhibitor cocktail of these five miRNAs, respectively. Inhibition of these five miRNAs resulted in the most remarkable upregulation of linc-ROR and SOX9 expression (Fig. 3b). Concomitantly, expression levels of stemness-associated genes were also decreased by miR-145 whereas the cocktail of miRNA inhibitors promoted their expression (Fig. 3c). Thus, these five miRNAs were pursued as candidates for studies in detail. Their predicted binding sites on linc-ROR transcript and SOX9 3′-UTR are shown in Fig. 3d and e. Moreover, these miRNAs exhibited decreased levels in the ESCC samples where linc-ROR expression was upregulated compared to their matched adjacent tissues (Additional file 2 Figure S1). Previous studies have demonstrated that miRNAs are present in the form of miRNA ribonucleoprotein complexes that contain Ago2, a key component of RNA-induced silencing complexes [39]. To validate the direct binding ability of these predicted miRNAs to linc-ROR and SOX9, RIP analysis was performed on EC9706 cells that were treated with the inhibitor cocktail of all candidate miRNAs or scramble control using Ago2 antibody. Linc-ROR and SOX9 were present in the Ago2 immunoprecipitates detected by qRT-PCR, and their levels were drastically reduced in Ago2 complexes purified from cells treated with miRNA inhibitor mixture (Fig. 3f), indicating that linc-RoR and SOX9 were recruited to the Ago2-containing–RNA-induced silencing complexes and functionally interacted with miRNAs. For further confirmation, we constructed a luciferase reporter containing Sox9 3′-UTR and co-transfected that with linc-ROR siRNA, cocktail of miRNA inhibitors, or miR-145 mimics into 293 T cells. Results of dual-luciferase reporter assay showed that linc-ROR repression or miR-145 overexpression significantly reduced the luciferase activities of the SOX9 3′-UTR reporter, whereas the treatment with the inhibitor cocktail of all candidate miRNAs increased the luciferase activity (Fig. 3g, bottom). The protein levels of SOX9 were also significantly decreased after knockdown of linc-ROR or overexpression of miR-145 (Fig. 3g, top). Collectively, these data suggest that linc-RoR and SOX9 share multiple regulatory miRNAs, and these candidate miRNAs negatively regulate linc-RoR and SOX9 directly.

### Candidate miRNAs suppress linc-ROR function

**Fig. 4:**
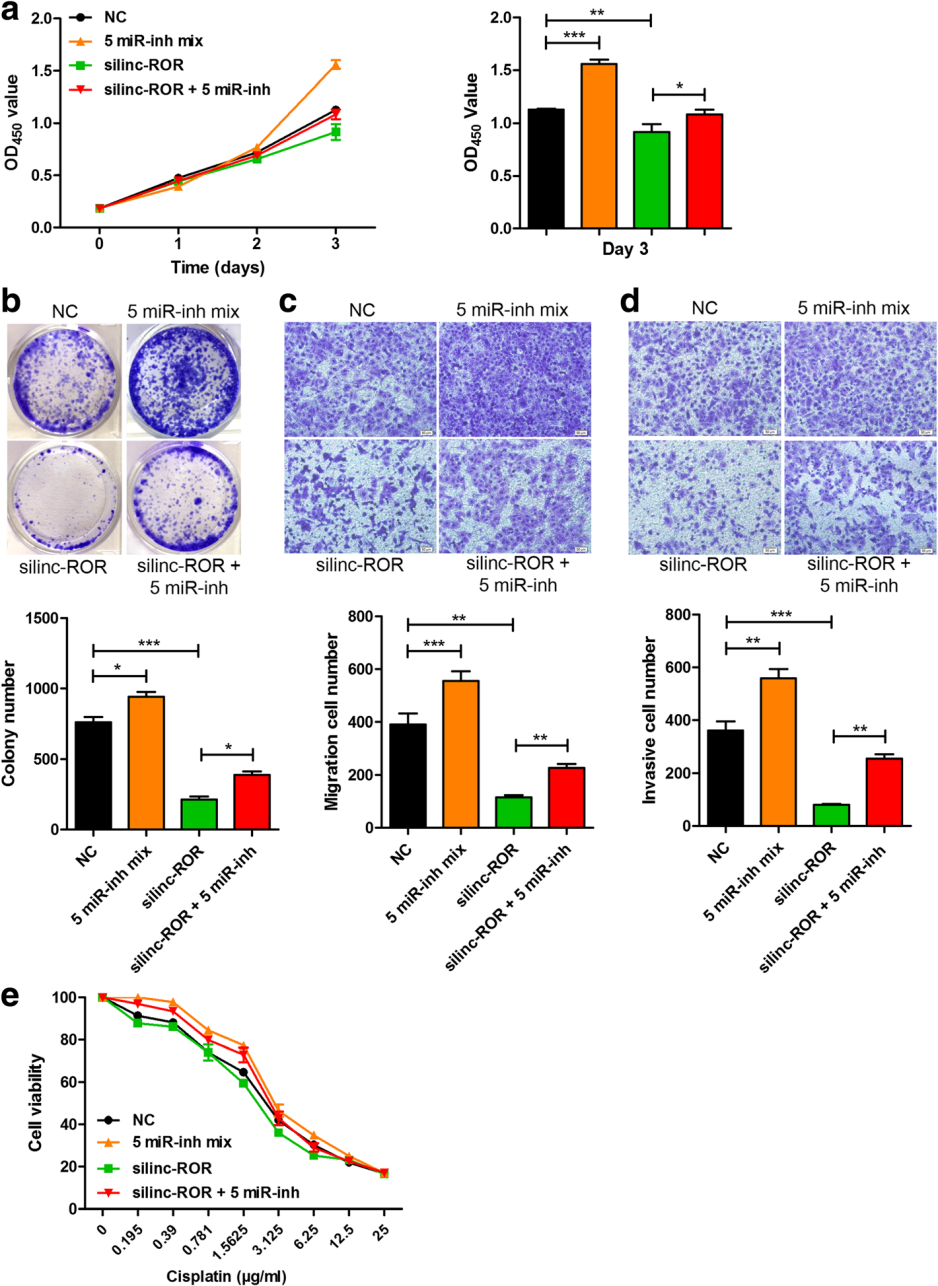
Candidate miRNAs suppress linc-ROR function. **a** EC9706 cells were transfected with inhibitor cocktail of five candidate miRNAs (5 miR-inh mix) with or without linc-ROR siRNA, and cell proliferation was determined using CCK8 assay. **b** Colony formation assay of EC9706 cells after co-transfection with inhibitor cocktail of all candidate miRNAs and linc-ROR siRNA. **c, d** Effect of antagonizing candidate miRNAs concomitant with linc-ROR knockdown on cell migration (**c**) and invasion (**d**) of EC9706 cells was assessed using Transwell assay. **e** Cell viability of EC9706 after co-transfection with inhibitor cocktail of all candidate miRNAs and linc-ROR siRNA was measured by CCK8 assay in the presence of indicated doses of cisplatin

To gain insight into the functional relevance of these candidate miRNAs, we examined the impacts of these miRNAs on cell proliferation, cell motility, and chemoresistance by antagonizing endogenous miR-15b, miR-33a, miR-129, miR-145, and miR-206 using specific antagomirs. CCK8 proliferation assay revealed that treatment with inhibitor cocktail of all candidate miRNAs promoted EC9706 cell proliferation, whereas co-transfection of miRNA inhibitor mixture and linc-ROR siRNA showed that the inhibition of these miRNAs could abrogate the suppressed cell proliferation caused by linc-ROR downregulation (Fig. 4a). Similarly, treatment with miRNA inhibitor cocktail promoted colony formation and partially abolished the inhibitory effect of linc-ROR knockdown (Fig. 4b). The migration ability (Fig. 4c) and invasive potential (Fig. 4d) of EC9706 cells were also increased following depletion of candidate miRNAs. Inhibition of these miRNAs in silinc-ROR-treated cells partially counteracted with linc-ROR silencing and promoted cell migration and invasion. Moreover, the increased chemosensitivity to cisplatin through knockdown of linc-ROR in EC9706 cells was abrogated upon inhibition of these candidate miRNAs (Fig. 4e). We further evaluated whether overexpression of candidate miRNA could potentiate the antitumor effects of linc-ROR repression. As expected, miR-145 functioned as tumor suppressor and reduced cell proliferation, colony formation, migration and invasion, and chemoresistance (Additional file 3 Figure S2). Combining silinc-ROR and miR-145 mimics synergistically inhibited colony formation and cell mobility in EC9706 cells compared to those of silinc-ROR or miR-145 mimics alone. Taken together, these observations suggest that these candidate miRNAs have potential antitumor effects through diminishing the function of linc-ROR on ESCC progression.

### SOX9 dominates linc-ROR/miRNA axis-mediated CSC-like properties

**Fig. 5:**
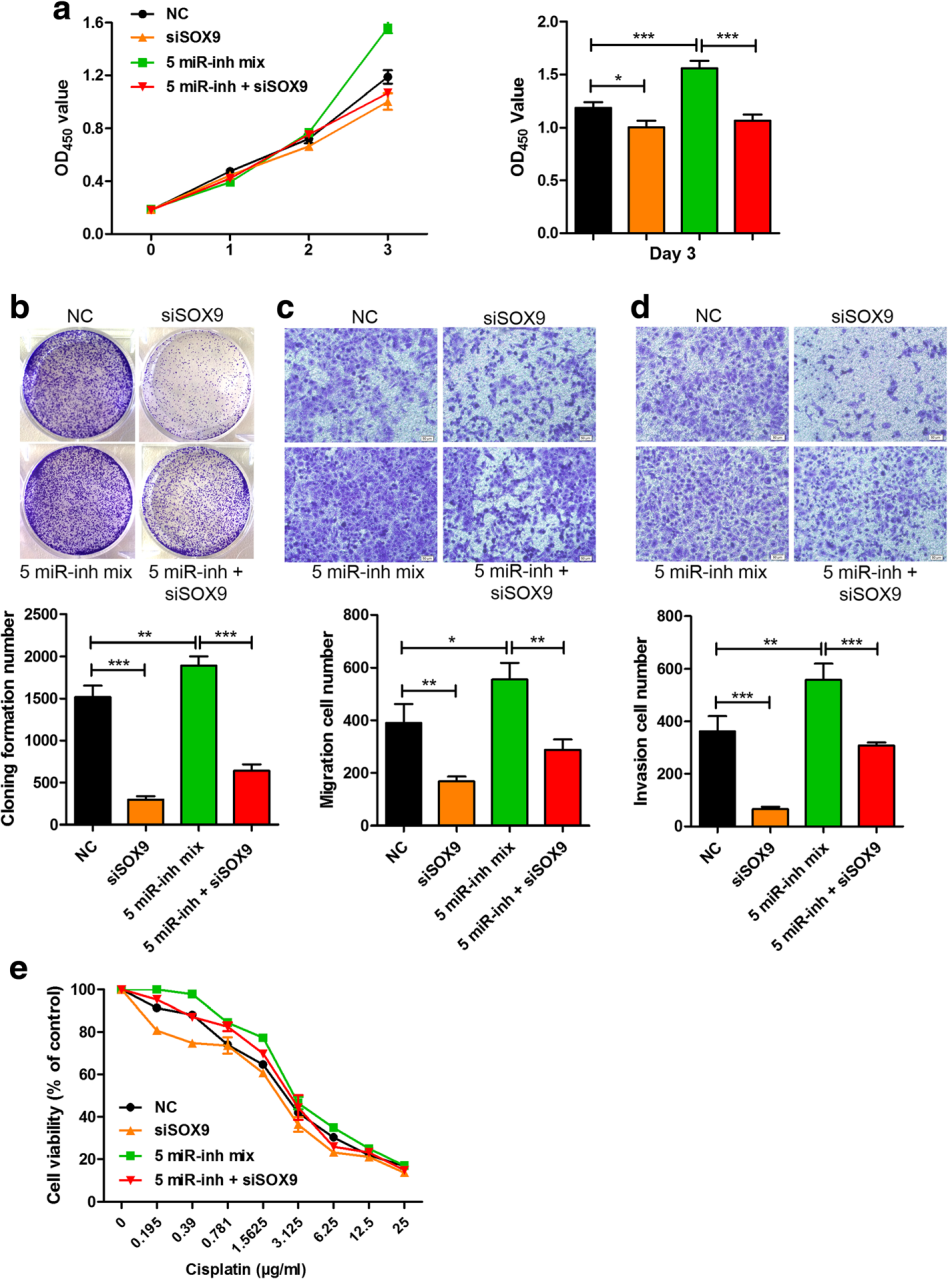
SOX9 dominates linc-ROR/miRNA axis-mediated CSC-like properties. **a** EC9706 cells were co-transfected with inhibitor cocktail of five candidate miRNAs (5 miR-inh mix) and SOX9 siRNA; then, cell proliferation was determined using CCK8 assay. **b** Colony formation assay of EC9706 cells after co-transfection with inhibitor cocktail of all candidate miRNAs and SOX9 siRNA. **c, d** Effect of antagonizing candidate miRNAs concomitant with SOX9 knockdown on cell migration (**c**) and invasion (**d**) of EC9706 cells was assessed using Transwell assay. **e** Cell viability of EC9706 after co-transfection with inhibitor cocktail of all candidate miRNAs and SOX9 siRNA was measured by CCK8 assay in the presence of indicated doses of cisplatin

To determine whether linc-ROR/miRNA axis-modulated proliferation, migration and invasion capacities, and chemoresistance were mediated by the target gene SOX9, we knocked down SOX9 expression using siRNA and investigated its effect on these phenotypes. Knockdown of SOX9 in EC9706 cells resulted in significantly reduced cell proliferation; cocktail of miRNA inhibitors-promoted cell proliferation was abrogated by additional repression of Sox9 (Fig. 5a). Similarly, silencing of SOX9 markedly reduced the enhancing effects of miRNA inhibitors on colony formation (Fig. 5b), migration and invasion capacities (Fig. 5c and d), and chemoresistance (Fig. 5e). These results collectively suggest that linc-ROR/miRNA axis-mediated acquisition of CSC properties is at least partly through SOX9 in ESCC.

### Targeting linc-ROR in ESCC for cancer therapy

**Fig. 6:**
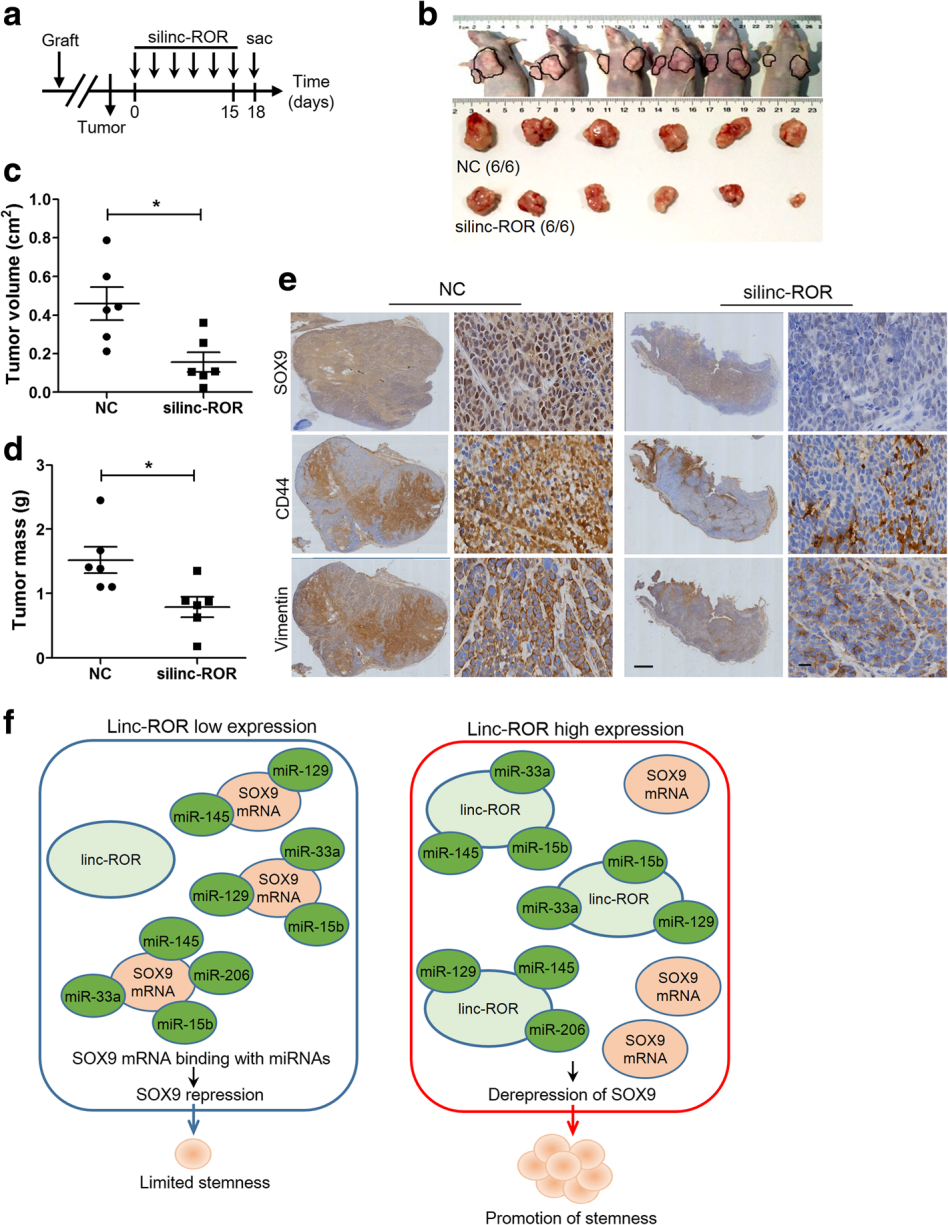
Targeting linc-ROR represses tumor growth and SOX9 expression in ESCC xenografts. **a** Experimental design for the linc-ROR targeted therapy using cholesterol-conjugated siRNA. **b** Tumors were excised from mice at the end of the experiment. **c, d** Tumor volume (**c**) and weight (**d**) were calculated as described in Materials and methods. **e** Representative immunohistochemistry staining of SOX9, CD44 and vimentin in ESCC xenografts after siRNA treatment. Scale bars, 1 mm (left) and 50 μm (right). **f** Proposed model for the role of the linc-ROR–miRNA–SOX9 regulatory network to promote ESCC progression

To provide the proof for the concept of the translational relevance of our findings, we performed in vivo therapeutic intervention studies using cholesterol-conjugated linc-ROR siRNA in established ESCC xenograft models. Mice bearing EC9706-derived xenografts were intratumorally injected with cholesterol-conjugated linc-ROR siRNA every 3 days for six times and sacrificed 3 days later (Fig. 6a). This treatment effectively inhibited tumor growth as measured by tumor volume and tumor weight compared to the control group (Fig. 6b–d). Furthermore, the level of SOX9 as well as CSC marker CD44 and mesenchymal marker vimentin in tumor xenografts was dramatically diminished by the treatment (Fig. 6e). These observations indicate that inhibition of linc-ROR expression decreases SOX9 activity in ESCC and as a result attenuates tumor growth.

## Discussion

Recent studies have demonstrated that linc-ROR dysregulation may be involved in carcinogenesis as well as cancer progression in several solid tumors [40]. In the current study, we revealed the positive correlation between linc-ROR and SOX9 expression in paired cancerous and noncancerous tissue samples, as well as a panel of ESCC cell lines. Accordingly, knockdown of linc-ROR suppressed the expression of SOX9. Loss-of-function approaches showed that linc-ROR depletion suppressed cell proliferation, cell motility, and chemoresistance in vitro and attenuated tumor growth in vivo, in line with recent evidences in other solid tumors such as breast [41] and pancreatic cancer [42]. Notably, we found that linc-ROR and SOX9 were overexpressed in tumorspheres compared with adherent cells, and linc-ROR disruption was sufficient to repress CSC marker expression in vitro and in vivo, as well as sphere-forming capacity, indicating they might coordinately regulate stemness in ESCC. Recent study also indicates that linc-ROR contributes to TGFβ-induced acquisition of CD133^+^ liver CSC and chemoresistance in hepatocellular cancer [43], although the underlying mechanisms have not yet been fully elucidated.

The long noncoding RNA–miRNA–mRNA regulatory network has been widely affirmed recently, in which long noncoding RNA functions as a ceRNA to interfere with miRNA and reduces their regulatory effect on target mRNA [44–47]. For example, *PTENP1* shares conserved miRNA seed target sites with *PTEN* 3′-UTR for the miR-17, miR-21, miR-214, miR-19, and miR-26 families, thereby modulating the expression of tumor suppressor gene *PTEN* [48]; lncRNA H19 regulates FOXM1 expression by competitively binding endogenous miR-342-3p, thus promoting cell proliferation and invasion in gallbladder cancer [49]. Similarly, linc-ROR can function as a ceRNA to regulate the expression of core transcription factors including OCT4, SOX2, and NANOG, whereas miR-145 is one of the related miRNAs which gained the most attention in pluripotent cells [15, 19, 40]. In the present study, we identified a novel interaction between linc-ROR and SOX9 mediated by multiple miRNAs, including miR-15b, miR-33a, miR-129, miR-145, and miR-206. Treatment with an inhibitor cocktail of all candidate miRNAs rescued linc-ROR knockdown-caused suppression of cell proliferation, cell motility, and chemoresistance. Linc-ROR/miRNA axis-mediated SOX9 activation might not be the only mechanism for regulating cancer stemness in ESCC, given that miR-145 has also been reported to enhance aggressive phenotypes by targeting NANOG in pancreatic and endometrial cancer [15, 35]. Linc-ROR also functions as an important inducer of EMT and metastasis through preventing mir-205 target genes from degradation in breast cancer [50]. However, the inhibition of Sox9 could abrogate malignant phenotypes induced by the cocktail of miRNA inhibitors, confirming that SOX9 at least partly modulates the linc-ROR/miRNA axis-mediated acquisition of CSC properties in ESCC. Therefore, aberrant expression of linc-ROR may serve as a novel mechanism underlying SOX9 deregulation, and the linc-ROR–miRNA–SOX9 regulatory network provides a novel vision to understand the oncogenic and tumor suppressor network puzzle.

In summary, the findings presented in this study revealed a linc-ROR–miRNA–SOX9 regulatory network in which linc-ROR modulated the deregulation of SOX9 at the posttrancriptional level through sequestering multiple SOX9-targeting miRNAs, including miR-15b, miR-33a, miR-129, miR-145, and miR-206, thereby granting CSC-like properties and promoting tumor progression (Fig. 6f). Identifying the precise role of linc-ROR allows us to better understand the pathogenesis and development of ESCC better and provides a potential precision therapeutic strategy for patients suffering from ESCC.

## Additional files

Additional file 1: **Table S1.** Clinical characteristics of patients with ESCC in this study. **Table S2.** Primer sequences for real-time PCR. **Table S3.** Predicted miRNA binding sites shared by linc-RoR and SOX9 3′-UTR. (DOCX 28 kb)

Additional file 2: Figure S1. Relative expression of candidate miRNAs in ESCC specimens compared with their matched adjacent tissues. +, upregulated linc-ROR in tumor compared with non-tumor counterpart; –, linc-ROR downregulation in tumor. (TIFF 156 kb)

Additional file 3: Figure S2. Overexpression of miR-145 potentiates the antitumor effects of linc-ROR knockdown. (A) EC9706 cells were transfected with miR-145 mimics with or without linc-ROR siRNA, and cell proliferation was determined using CCK8 assay. (B) Colony formation assay of EC9706 cells after cotransfectionwith miR-145 mimics and linc-ROR siRNA. (C, D) Effect of miR-145 overexpression concomitant with linc-ROR knockdown on cell migration (C) and invasion (D) of EC9706 cells was assessed using Transwell assay. (E) Cell viability of EC9706 after co-transfection with miR-145 mimics and linc-ROR siRNA was measured by CCK8 assay in the presence of indicated doses of cisplatin. (TIFF 3007 kb)

## Abbreviation

3′-UTR: 3′-untranslated region
ceRNA: Competitive endogenous RNA
CSC: Cancer stem cell
ESCC: Esophageal squamous cell carcinoma
linc-ROR: Long intergenic non-protein coding RNA, regulator of reprogramming
SOX9: Sex determining region Y box 9
