# Supplementary material for: Linc-ROR promotes esophageal squamous cell carcinoma progression through the derepression of SOX9
Source: J Exp Clin Cancer Res. 2017 Dec 13;36:182. doi: 10.1186/s13046-017-0658-2 (PMC5727696; doi:10.1186/s13046-017-0658-2)
Supplement: Supplementary file 1 — Table S1. Clinical characteristics of patients with ESCC in this study. Table S2. Primer sequences for real-time PCR. Table S3. Predicted miRNA binding sites shared by linc-RoR and SOX9 3′-UTR. (DOCX 28 kb) [file 13046_2017_658_MOESM1_ESM.docx]

**Supplementary Table 1.** Clinical characteristics of patients with ESCC in this study.

| Characteristics | Number of cases |
| --- | --- |
| **Sex** |  |
| Male | 9 |
| Female | 5 |
| **Age at diagnosis** |  |
| < 60 | 4 |
| ≥ 60 | 10 |
| **Tumor location** |  |
| Upper | 1 |
| Middle | 8 |
| Lower | 5 |
| **Tumor Size** |  |
| < 5cm | 6 |
| ≥ 5cm | 8 |
| **Grade of differentiation** |  |
| well | 0 |
| moderate | 12 |
| poor | 2 |
| **Depth of invasion** |  |
| T1 | 0 |
| T2 | 6 |
| T3 | 7 |
| T4 | 1 |
| **Lymph node metastasis** |  |
| N0 | 13 |
| N1 | 1 |
| **TNM stage** |  |
| I/IIA | 9 |
| IIB/III/IV | 5 |

**Supplementary Table 2.** Primer sequences for real-time PCR.

| Gene symbol | Primer sequences |
| --- | --- |
| Linc-ROR | For：5′-TTCAGTTCCCTAAAGTCACCC-3′  Rev：5′-GTCCTTCTAAGCCTCTGTTGC-3′ |
| SOX9 | For：5′-TCCTCAGGCTTTGCGATTT-3′  Rev：5′-TGCTCGGGCACTTATTGG-3′ |
| KLF4 | For：5′-GAAATTCGCCCGCTCAGATGAACT-3′  Rev：5′-TCTTCATGTGTAAGGCGAGGTGGT-3′ |
| NANOG | For：5′-AATACCTCAGCCTCCAGCAGATG-3′  Rev：5′-TGCGTCACACCATTGCTATTCTTC-3′ |
| OCT4 | For：5′-TATTCAGCCAAACGACCATCT-3′  Rev：5′-ATTATTGTCAGCTTCCTCCAC-3′ |
| SOX2 | For：5′-AGATAAACATGGCAATCAAAATG-3′  Rev：5′-CAACGGTGTCAACCTGCATGGCC-3′ |
| CD44 | For：5′-ACTGTTATATCAGAGGAGTAGGAGA-3′  Rev：5′-ACAGCACAAGAATGAACAATGG-3′ |
| CD133 | For：5′-CATTGACTTGGTGCTGTTGA-3′  Rev：5′-CTGCGTGAATATGCTGTAGG-3′ |
| GAPDH | For：5′-GCACCGTCAAGGCTGAGAAC-3′  Rev：5′-TGGTGAAGACGCCAGTGGA-3′ |

**Supplementary Table 3.** Predicted miRNA binding sites shared by linc-RoR and SOX9 3′-UTR.

| miRNA | Position of miRNA bingding sites | |
| --- | --- | --- |
|  | Linc-ROR | SOX9 3′-UTR |
| hsa-miR-15b-5p | 2121-2126 | 860-866 |
| hsa-miR-33a-5p | 2395-2400 | 1549-1556 |
| hsa-miR-33b-5p | 2395-2400 | 1549-1556 |
| hsa-miR-129-5p | 2351-2356 | 1155-1161 |
| hsa-miR-138-5p | 971-977 | 1082-1089 |
| hsa-miR-145-5p | 665-670 | 1402-1409 |
| hsa-miR-185-5p | 1157-1163 | 1941-1947 |
| hsa-miR-186-5p | 312-316 | 1164-1170 |
| hsa-miR-206 | 1595-1599 | 1103-1110 |
| hsa-miR-497-5p | 2121-2126 | 860-866 |
| hsa-miR-539-5p | 2322-2328 | 1572-1578 |
| hsa-miR-5195-3p | 821-826 | 1402-1409 |

**Supplementary Figure Legends**

Supplementary Figure 1: Relative expression of candidate miRNAs in ESCC specimens compared with their matched adjacent tissues. +, upregulated linc-ROR in tumor compared with non-tumor counterpart; –, linc-ROR downregulation in tumor.

Supplementary Figure 2: Overexpression of miR-145 potentiates the antitumor effects of linc-ROR knockdown. (A) EC9706 cells were transfected with miR-145 mimics with or without linc-ROR siRNA, and cell proliferation was determined using CCK8 assay. (B) Colony formation assay of EC9706 cells after co-transfection with miR-145 mimics and linc-ROR siRNA. (C, D) Effect of miR-145 overexpression concomitant with linc-ROR knockdown on cell migration (C) and invasion (D) of EC9706 cells was assessed using Transwell assay. (E) Cell viability of EC9706 after co-transfection with miR-145 mimics and linc-ROR siRNA was measured by CCK8 assay in the presence of indicated doses of cisplatin.
