# Supplementary figures and images for: Linc-ROR promotes esophageal squamous cell carcinoma progression through the derepression of SOX9
Source: J Exp Clin Cancer Res. 2017 Dec 13;36:182. doi: 10.1186/s13046-017-0658-2 (PMC5727696; doi:10.1186/s13046-017-0658-2)

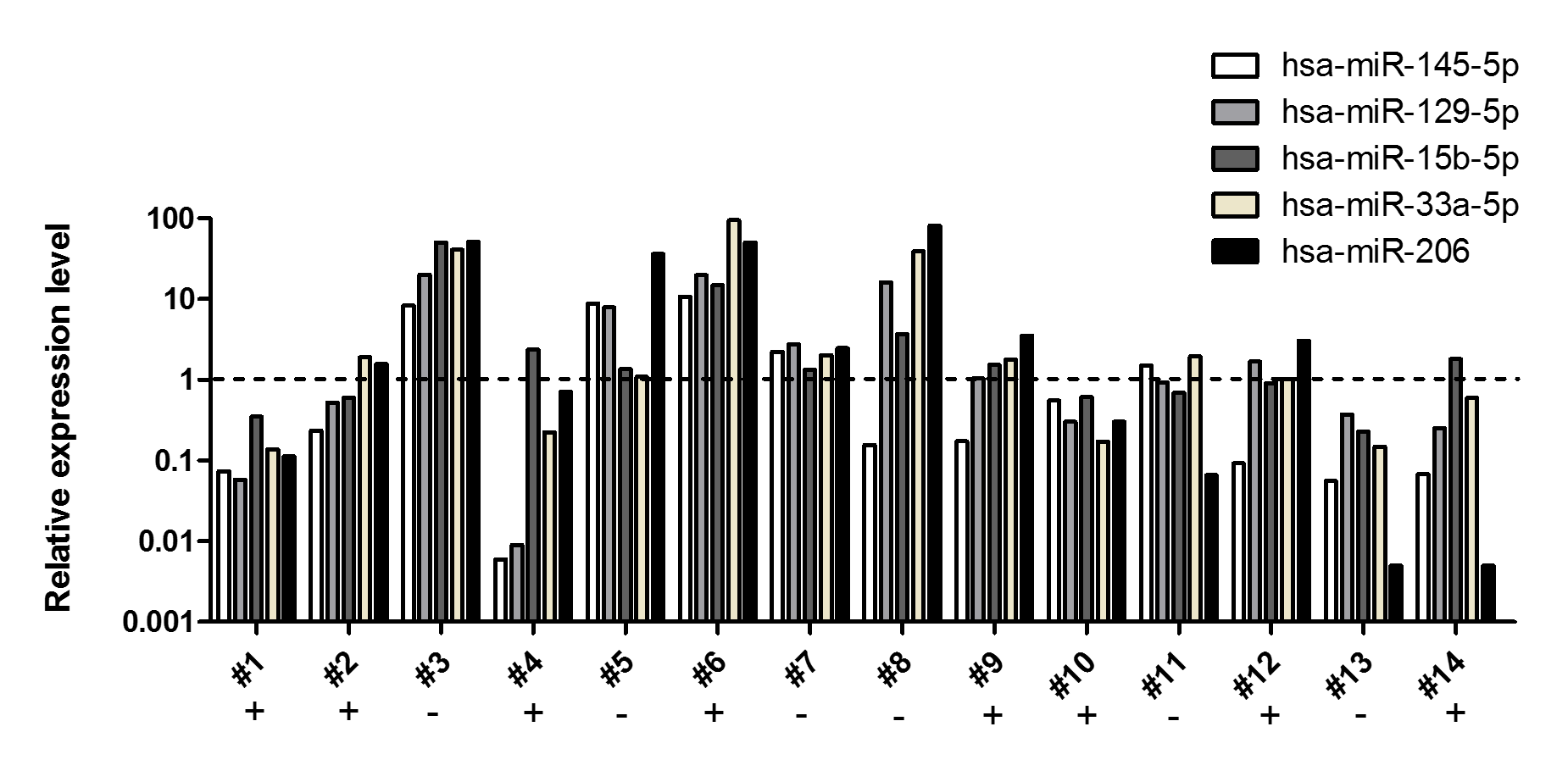

Supplement: Supplementary file 2 — Relative expression of candidate miRNAs in ESCC specimens compared with their matched adjacent tissues. +, upregulated linc-ROR in tumor compared with non-tumor counterpart; –, linc-ROR downregulation in tumor. (TIFF 156 kb) [file 13046_2017_658_MOESM2_ESM.tif]

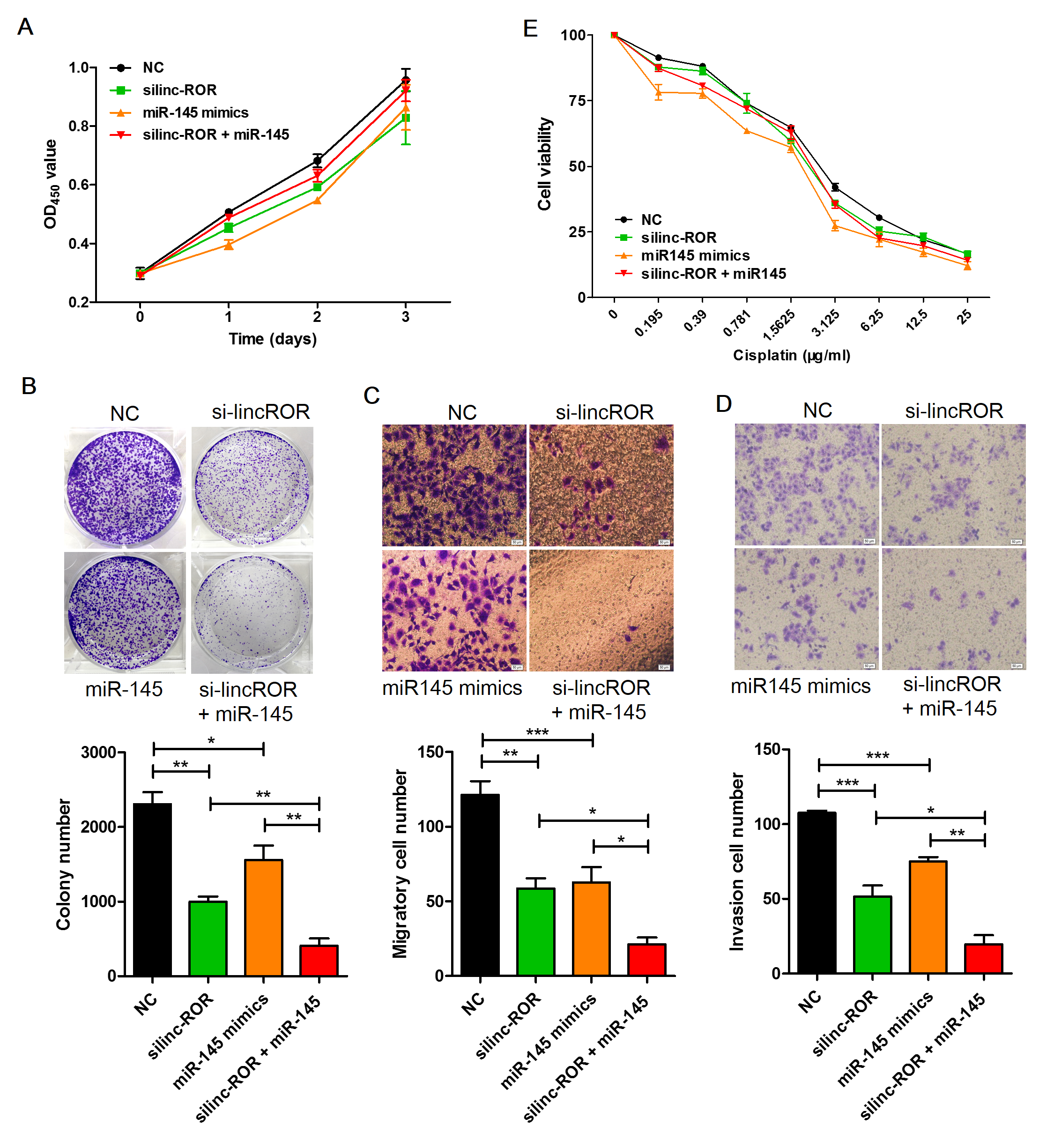

Supplement: Supplementary file 3 — Overexpression of miR-145 potentiates the antitumor effects of linc-ROR knockdown. (A) EC9706 cells were transfected with miR-145 mimics with or without linc-ROR siRNA, and cell proliferation was determined using CCK8 assay. (B) Colony formation assay of EC9706 cells after cotransfectionwith miR-145 mimics and linc-ROR siRNA. (C, D) Effect of miR-145 overexpression concomitant with linc-ROR knockdown on cell migration (C) and invasion (D) of EC9706 cells was assessed using Transwell assay. (E) Cell viability of EC9706 after co-transfection with miR-145 mimics and linc-ROR siRNA was measured by CCK8 assay in the presence of indicated doses of cisplatin. (TIFF 3007 kb) [file 13046_2017_658_MOESM3_ESM.tif]
